# Performance of the tuberculin skin test and interferon-γ release assay for detection of tuberculosis infection in immunocompromised patients in a BCG-vaccinated population

**DOI:** 10.1186/1471-2334-9-207

**Published:** 2009-12-15

**Authors:** Eun Young Kim, Ju Eun Lim, Ji Ye Jung, Ji Young Son, Kyung Jong Lee, Yoe Wun Yoon, Byung Hoon Park, Jin Wook Moon, Moo Suk Park, Young Sam Kim, Se Kyu Kim, Joon Chang, Young Ae Kang

**Affiliations:** 1 Department of Internal Medicine, Yonsei University College of Medicine, 250 Seongsanno, Seodaemun-gu, Seoul, Republic of Korea

## Abstract

**Background:**

Interferon-γ release assay (IGRA) may improve diagnostic accuracy for latent tuberculosis infection (LTBI). This study compared the performance of the tuberculin skin test (TST) with that of IGRA for the diagnosis of LTBI in immunocompromised patients in an intermediate TB burden country where BCG vaccination is mandatory.

**Methods:**

We conducted a retrospective observational study of patients given the TST and an IGRA, the QuantiFERON-TB Gold In-Tube (QFT-IT), at Severance Hospital, a tertiary hospital in South Korea, from December 2006 to May 2009.

**Results:**

Of 211 patients who underwent TST and QFT-IT testing, 117 (55%) were classified as immunocompromised. Significantly fewer immunocompromised than immunocompetent patients had positive TST results (10.3% vs. 27.7%, p 0.001), whereas the percentage of positive QFT-IT results was comparable for both groups (21.4% vs. 25.5%). However, indeterminate QFT-IT results were more frequent in immunocompromised than immunocompetent patients (21.4% vs. 9.6%, p 0.021). Agreement between the TST and QFT-IT was fair for the immunocompromised group (κ = 0.38), but moderate agreement was observed for the immunocompetent group (κ = 0.57). Indeterminate QFT-IT results were associated with anaemia, lymphocytopenia, hypoproteinemia, and hypoalbuminemia.

**Conclusion:**

In immunocompromised patients, the QFT-IT may be more sensitive than the TST for detection of LTBI, but it resulted in a considerable proportion of indeterminate results. Therefore, both tests may maximise the efficacy of screening for LTBI in immunocompromised patients.

## Background

Tuberculosis (TB) is the single most important cause of death due to infectious disease worldwide, resulting in ~1.8 million deaths annually [1]. For effective and efficient control of TB, rapid diagnosis and treatment for active TB patients is the mainstay of national TB programs in developing countries. However, treatment of active TB is not sufficient to eliminate the disease, because individuals with latent TB infection (LTBI) outnumber those with active TB, and LTBI can progress to active disease at any time [2]. For this reason, the diagnosis and treatment of individuals with LTBI who are at higher risk of developing active TB is an important goal of TB control programs in developed countries [3]. However, diagnosis of LTBI is problematic because the tuberculin skin test (TST), which has been widely used for centuries, has several limitations. False-positive results caused by exposure to nontuberculosis mycobacteria or prior Bacille Calmette-Guerin (BCG) vaccination, false-negative results due to cutaneous anergy with underlying immunosuppression, interobserver variability, and the booster effect reduce the efficiency of a strategy of targeted use of the TST and treatment of LTBI [4-7]. To overcome the limitations of the TST, some countries have implemented interferon-γ release assays (IGRAs) as part of their national TB programs [8-11]. IGRA tests are based on the release of interferon-γ in blood samples after stimulation in vitro with *M. tuberculosis *(MTB)-specific antigens [12]. IGRA tests are highly specific for diagnosing active TB and LTBI in BCG-vaccinated individuals [13,14] and are more sensitive than the TST for the diagnosis of active TB in immunocompromised patients [15,16]. In addition to their improved diagnostic accuracy, IGRA tests have operational advantages over the TST [17]. Therefore, IGRA tests are expected to improve the effectiveness of TB control programs in many countries with low prevalence of TB. However, the use of IGRA tests for the diagnosis of LTBI in immunocompromised patients is still limited, although new data are emerging [11,18-20]. To eliminate TB, it is essential to improve the efficiency of diagnosis and treatment of LTBI among immunocompromised patients at high risk for developing active TB [17].

In South Korea, treatment of LTBI has been recommended only for children aged <6 years who have been exposed to TB, for HIV-infected individuals, for patients receiving tumour necrosis factor-α inhibitors after diagnosis of LTBI using the TST [21]. However, LTBI in immunocompromised patients has not been adequately studied in South Korea using the newer IGRA tests. The aim of this study was to evaluate the performance of the TST and IGRA in the diagnosis of LTBI infection in immunocompromised patients compared to immunocompetent patients in South Korea, where the incidence of active TB is intermediate (70-90/100,000 per year) and BCG vaccination is mandatory [1].

## Methods

### Study population

Patients tested for TB infection with the TST and an IGRA, the QuantiFERON-TB Gold In-Tube (QFT-IT), were included in the study. Patients were tested at Severance Hospital (Seoul, Republic of Korea), a university-affiliated tertiary care referral hospital, between December 2006 and May 2009. We reviewed patients' medical records, microbiologic results, other laboratory results, and radiographic results. Patients who were diagnosed with active TB during the study period or who had previously been treated for TB were excluded to enable evaluation of LTBI. The protocol for this study was approved by the Ethical Review Committee of Severance Hospital. We included 211 patients who underwent both the TST and QFT-IT. Most (197) participants were tested for suspicion of active TB during a clinical work up by attending physicians, nine patients underwent the tests for screening before tumour necrosis factor-α inhibitors were administered, and five patients before transplantation.

The definition of an immunocompromised condition included the following: 1) diabetes mellitus 2) undergoing chemotherapy for an underlying malignancy at the time of TST and QFT-IT testing, 3) received either a solid organ transplant or bone marrow transplant, 4) end-stage renal disease and on renal replacement therapy, 5) advanced liver cirrhosis with Child-Pough class C, 6) seropositivity for human immunodeficiency virus, 7) daily administration of systemic corticosteroids (at least 15 mg of prednisone per day for more than one month or combination therapy with low dose corticosteroids and other immunosuppressants including azathioprine, mycophenolate, methotrexate, cyclosporine, or cyclophosphamide).

### TST

The TST was performed by injecting a 2-TU dose of purified protein derivative RT23 (Statens Serum Institut, Copenhagen, Denmark) intradermally into the forearm using the Mantoux technique [22]. The criterion for a positive TST result was an induration 10 mm or greater in size occurring 48-72 h after injection.

### QFT-IT

The QFT-IT test was performed in the Immunology Laboratory at Severance Hospital according to the recommendations of the manufacturer (Cellestis Ltd; Carnegie, Australia) Briefly, 1 ml blood was drawn directly into each of three evacuated blood collection tubes: one containing heparin alone (the nil tube, or the negative control); one containing the T cell mitogen phytohemagglutinin (the mitogen tube, or the positive control); and one containing the MTB-specific antigens ESAT-6, CFP-10, and TB7.7 (the TB antigen tube). After mixing, the tubes were incubated upright for 20 h at 37°C before plasma was harvested and then were stored frozen at -20°C until further analysis within 5 days. The concentration of interferon-γ in each plasma sample was determined using QFT ELISA. Results were calculated using the QFT-IT software provided by the manufacturer.

### Statistical analysis

Data are expressed as number (percentage) or median and interquartile range (because the majority of data did not follow a normal distribution). Categorical variables were analysed using Pearson's χ^2 ^test or Fisher's exact test; continuous variables were analysed using Mann-Whitney U tests. The concordance between the TST and QFT-IT test results was assessed using κ coefficients and was interpreted according to the Landis and Koch's classification [23].

All tests of significance were two-tailed; *P *< 0.05 was considered significant. SPSS v. 11.5 (SPSS Inc., Chicago, IL, USA) was used for statistical analyses.

## Results

### Baseline clinical characteristics of participants

A total of 211 participants underwent both TST and QFT-IT testing for TB infection. Baseline characteristics of participants are shown in Table 1. Of the 211 participants, 117 (55%) were defined as immunocompromised because of underlying disease: 40 patients undergoing treatment with immunosuppressive drugs, 23 with diabetes, 28 with solid cancer undergoing anti-cancer chemotherapy, 7 with haematologic malignancies and chemotherapy, 11 with organ transplantation, 6 with end-stage renal disease, 1 with advanced liver cirrhosis, and 1 with HIV infection. No significant differences were observed between the immunocompromised and immunocompetent groups in terms of the distribution of age, sex, body mass index, history of smoking, positive history of recent TB contact, presence of a TB scar on simple chest radiographs, white blood cell count, number of platelets, or serum level of cholesterol. However, in laboratory findings, the concentration of haemoglobin, number of lymphocytes, and serum levels of protein and albumin were significantly lower in the immunocompromised group than in the immunocompetent group (Table 1).

**Table 1:** Baseline characteristics of 211 participants

Characteristic	All participants *n *= 211	Immunocompetent *n *= 94	Immunocompromised *n *= 117	*p*
Age, median (range)	55 (19-98)	53 (19-98)	55 (19-93)	0.13
Male/female	97/114	45/49	52/65	0.62
BMI (median, IQR)	21.6 (19.5-24.5)	21.2 (18.8-23.9)	22.2 (19.9-25.0)	0.09
History of smoking	34 (16)	14 (15)	20 (17)	0.19
History of recent TB contact				
Negative	181 (85.8)	85 (90.4)	96 (82.1)	
Positive	2 (0.9)	2 (2.1)	0 (0)	0.22*
Unknown	28 (13.3)	7 (7.4)	21 (17.9)	
Presence of TB scar on chest X-ray	7 (3.3)	5 (5.3)	2 (1.7)	0.25
Laboratory findings (median, IQR)				
WBC (10^3^/uL)	7350 (5480-10570)	7260 (5900-9380)	7430 (5030-11280)	0.89
Haemoglobin (g/dL)	11.1 (9.5-12.7)	11.9 (10.75-13.4)	10.6 (9.2-12.0)	< 0.001
Platelets (10^3^/uL)	272 (196-380)	277 (210-394)	266 (170-379)	0.16
Lymphocytes (10^3^/uL)	1410 (860-1890)	1670 (1155-2085)	1230 (755-1700)	< 0.001
Total protein (g/dL)	6.8 (6.1-7.38)	7.2 (6.7-7.6)	6.5 (5.7-7.0)	< 0.001
Albumin (g/dL)	3.6 (2.9-4.2)	4.0 (3.2-4.4)	3.2 (2.8-3.9)	< 0.001
Cholesterol (mg/dL)	152 (122-184)	160 (128-184)	149 (117-182)	0.14
Disease associated with immunosuppression	117 (55)			
Diabetes	23 (11)			
Solid cancer treated with anticancer chemotherapy	28 (13)			
Haematologic malignant disease	7 (3)			
Transplantation	11(5)			
ESRD on renal replacement therapy	6 (3)			
Liver cirrhosis, Child-Pough class C	1 (0.5)			
HIV infection	1 (0.5)			
Treated with immunosuppressive drugs†	40 (19)			

Data are presented as numbers (percentages) unless otherwise indicated.

BMI, body mass index; ESRD, end-stage renal disease; IQR, interquartile range; TB, tuberculosis; WBC, white blood cells.

*p 0.22 for the difference in the history of recent TB contact, excluding participants whose history of recent TB contact was unknown.

†Patients receiving systemic corticosteroids (at least 15 mg of prednisone per day for more than one month) or combination therapy with low dose corticosteroids and other immunosuppressants including azathioprine, mycophenolate, methotrexate, cyclosporine, or cyclophosphamide.

### Performance of the TST and QFT-IT in immunocompetent and immunocompromised patients

Overall, the TST showed positive results in 38 patients (18%) and negative results in 173 patients (82%), whereas the QFT-IT showed positive results in 49 patients (23.2%) and negative results in 128 patients (60.7%). Thirty-four patients (16.1%) had indeterminate QFT-IT assay results. The proportion of positive TST results was higher in the immunocompetent group (27.7%) than in the immunocompromised group (10.3%, *p *0.001). For the QFT-IT, the proportion of positive results was comparable for both groups (25.5% vs 21.4%, *p *0.068); however, the proportion of indeterminate results was higher in the immunocompromised group than in the immunocompetent group (21.4% vs 9.6%, *p *0.021; Table 2).

**Table 2:** Results of the TST and QFT-IT in 211 participants who underwent evaluation for tuberculosis infection

Assay result	All participants *n *= 211	Immunocompetent *n *= 94	Immunocompromised *n *= 117
TST			
Positive*	38 (18)	26 (27.7)	12 (10.3)
Negative	173 (82)	68 (72.3)	105 (89.7)
QFT-IT			
Positive†	49 (23.2)	24 (25.5)	25 (21.4)
Negative	128 (60.7)	61 (64.9)	67 (57.3)
Indeterminate‡	34 (16.1)	9 (9.6)	25 (21.4)

Data are presented as numbers (percentages) unless otherwise indicated.

QFT-IT, QuantiFERON-TB Gold In-Tube; TST, tuberculin skin test.

*p 0.001 for the difference in the number of positive TST results between the groups.

†*p *0.068 for the difference of QFT-IT results between the groups.

‡*p *0.021 for the difference in the proportion of indeterminate QFT-IT results between the groups.

### Concordance between the TST and QFT-IT in immunocompetent and immunocompromised patients

Table 3 shows the concordance between the TST and QFT-IT. The TST and QFT-IT assays showed moderate agreement in the immunocompetent group (κ = 0.57, *p *0.001). However, agreement was fair in the immunocompromised group (κ = 0.38, *p *0.001). Agreement between the TST and QFT-IT did not show a remarkable change (κ = 0.40, *p *0.001) when we used a 5-mm induration cut off for the TST in the immunocompromised group.

**Table 3:** Diagnostic agreement of TST and QFT-IT (κ) *

	QFT-IT +	QFT-IT -	Total	κ
All participants				0.48
TST +	26 (14.7)	11 (6.2)	37	
TST -	23 (13.0)	117 (66.1)	140	
Total	49	128	177	
Immunocompetent group				0.57
TST +	17 (20.0)	8 (9.4)	25	
TST -	7 (8.2)	53 (62.4)	60	
Total	24	61	85	
Immunocompromised group				0.38
TST+	9 (9.8)	3 (3.3)	12	
TST -	16 (17.4)	64 (69.6)	80	
Total	25	67	92	

Immunocompromised group*				0.40
TST+*	14 (15.2)	11 (12.0)	25	
TST -*	11 (12.0)	56 (60.9)	67	
Total	25	67	92	

Data are presented as numbers (percentages) unless otherwise indicated.

QFT-IT, QuantiFERON-TB Gold In-Tube; TST, tuberculin skin test.

*Agreement between the two tests with a 5-mm induration cut off for the TST in the immunocompromised group.

### Clinical characteristics of patients with indeterminate results for the QFT-IT

Table 4 shows the characteristics of 34 patients with indeterminate QFT-IT results and the remaining 117 patients with determinate results. Immunosuppressive conditions, low levels of haemoglobin, leukocytosis, lymphocytopenia, hypoproteinemia, and hypoalbuminemia were more frequently observed in patients with indeterminate than determinate results. Only one patient (3%) with indeterminate QFT-IT results showed a positive TST result. In the multivariate analysis, lymphocytopenia (odds ratio [OR] 0.998, 95% confidence interval [CI] 0.997-0.998) and cholesterol level (OR 1.01, 95% CI 1.002-1.024) were independently associated with the indeterminate QFT-IT results. Hypoalbuminemia showed marginal significance (OR 0.288, 95% CI 0.080-1.040, *p *0.057).

**Table 4:** Characteristics of patients with indeterminate and determinate QFT-IT results

Characteristic	Indeterminate *n *= 34	Determinate *n *= 177	*p*
Age, median (range)	61 (20-98)	54 (19-85)	0.52
Male/female	13/21	84/93	0.32
BMI, (median, IQR)	21.6 (20.8-25.4)	21.5 (19.2-24.4)	0.28
Recent TB contact			1.0*
Negative	25 (73.5)	156 (88.1)	
Positive	0 (0)	2 (1.1)	
Unknown	9 (26.5)	19 (10.7)	
Presence of TB scar on chest X-ray	0 (0)	7 (4.0)	0.24
History of smoking	4 (12)	30 (17)	0.44
Hypertension	12 (35)	37 (21)	0.07
Positive results of TST	1 (3)	49 (28)	0.002
Immunosuppressive condition	25 (74)	92 (52)	0.02
Diabetes	3	20	
Solid cancer treated with anticancer chemotherapy	2	26	
Haematologic malignant disease	2	5	
Transplantation	4	7	
ESRD on renal replacement therapy	1	5	
Liver cirrhosis, Child-Pough class C	0	1	
HIV infection	0	1	
Treated with immunosuppressive drugs	13	27	
Laboratory findings, (median, IQR)			
WBC (10^3^/uL)	10 695 (4840-16275)	7160 (5510-9660)	0.04
Haemoglobin (g/dL)	9.9 (8.9-10.9)	11.5 (9.8-13.1)	< 0.001
Platelets (10^3^/uL)	317 (214-399)	267 (196-376)	0.43
Lymphocytes (10^3^/uL)	780 (435-1582)	1500 (1005-1925)	< 0.001
Total protein (g/dL)	6.45 (5.3-6.8)	6.9 (6.2-7.4)	< 0.001
Albumin (g/dL)	3.0 (2.7-3.2)	3.8 (3.1-4.2)	< 0.001
Cholesterol (mg/dL)	138 (109-182)	156 (125-184)	0.12

Data are presented as numbers (percentages) unless otherwise indicated.

MI, body mass index; ESRD, end-stage renal disease; IQR, interquartile range; QFT-IT, QuantiFERON-TB Gold In-Tube; TB, tuberculosis; TST, tuberculin skin test; WBC, white blood cells.

*p 1.00 for the difference in history of recent TB contact, except where unknown.

## Discussion

Among our 211 participants, including 117 (55%) immunocompromised patients, the number of patients with positive QFT-IT results was comparable between the immunocompetent and immunocompromised groups (25.5% vs 21.4%, respectively), whereas the TST detected significantly less LTBI among the immunocompromised group than the immunocompetent group (27.7% vs 10.3%). In addition, for the 177 patients with determinate QFT-IT and TST results, the proportions of positive results for the TST and QFT-IT were significantly different among the immunocompromised group (27.2% vs 13.0%, respectively) but were comparable among the immunocompetent group (29.4% vs 28.2%, respectively). These findings suggest that the sensitivity of the QFT-IT for the diagnosis of LTBI infection might be higher than that of the TST in immunocompromised patients. The results of our study are consistent with those of previous studies reporting the higher sensitivity of the IGRA test compared to the TST [18,20,24] for the diagnosis of TB infection. The false-negative results of the TST may result from poor cell-mediated immunity [22]; concerns have frequently been raised regarding the effects of cutaneous anergy in immunosuppressive conditions on the results of the TST, and this issue has been considered a weak point of the TB elimination strategy [6,17]. Therefore, the higher sensitivity of in vitro IGRA tests for the diagnosis of LTBI in immunocompromised patients could strengthen the targeted testing strategy and treatment of LTBI.

The moderate concordance between the TST and QFT-IT (κ = 0.57, p < 0.001) in the immunocompetent group in contrast with the fair concordance of the TST and QFT-IT (κ = 0.38, p 0.001) in the immunocompromised group revealed that more patients had TST negative/QFT-IT positive results in the immunocompromised group. Considering the high specificity of the QFT-IT for MTB infection [12], these results suggest that a substantial proportion of immunocompromised patients with LTBI may be missed by the TST. However, half of the discordant results of the immunocompetent group resulted from a TST positive/QFT-IT negative, raising concern for the low sensitivity of the QFT-IT. Considering that the TB infection rate in 20- to 29-year-old Koreans was 59% in 1995 [25] and more than half of the participants in our study were > 50 years, there was a possibility for false negative QFT-IT results estimating latent TB infection. Furthermore, a large contact investigation of a South Korean high-school outbreak showed that more than 20% of the contacts with a positive on the TST with more than 20 mm induration, but with a negative QuantiFERON-TB Gold assay, were diagnosed with active TB in BCG vaccination circumstances[26]. Therefore, it is difficult to disregard the possibility of a true M. TB infection among the three immunocompromised patients with a TST positive/QFT-IT negative in our study.

Although our results suggest that the QFT-IT improves the accuracy of the diagnosis of LTBI in immunocompromised groups, the QFT-IT had a considerable proportion of indeterminate results in the immunocompromised group (21.4%). In this study, indeterminate results for the QFT-IT occurred at a relatively high rate compared to in previous studies, which ranged from 1-21% [27-29]. The relatively high proportion of indeterminate results could be explained by either local or systemic inflammatory conditions affecting the participants in our study when the QFT-IT tests were performed in routine clinical practice. Laboratory findings of lymphocytopenia but relative leukocytosis associated with indeterminate QFT-IT results suggest that the immunocompromised patients had a combination of inflammatory and immunosuppressive conditions, along with hypoalbuminemia, which indicates poor nutritional status. These results are consistent with previously reported risk factors for indeterminate QFT-IT results [29]. It is interesting that only one patient with indeterminate QFT-IT results had a positive TST result, with a 12-mm induration; the other 33 patients showed negative TST results without any induration. These results suggest that most of these patients showed an anergic response to the stimulus; however, not all patients with indeterminate QFT-IT results had negative TST results in previous studies [20], which implies that individuals with an indeterminate QFT-IT result may in fact have LTBI.

In South Korea, the TB infection rate in 20- to 29-year-olds was 59% in 1995, and the expected prevalence of LTBI in all Koreans is ~30% [14,25]. Treatment of LTBI is recommended only for exposed children aged <6 years, HIV-infected individuals, and patients receiving tumour necrosis factor-α inhibitors only after diagnosis of LTBI infection by TST [21]. Although the treatment of LTBI has not been recommended for other immunocompromised patients, including individuals who have undergone transplantation or immunosuppressive treatments such as steroids, or those who have end-stage renal disease or malignancy treated with anti-cancer chemotherapy, physicians have become increasingly interested in the treatment of LTBI. Although the prevalence of active TB is decreasing, the number of immunocompromised hosts is increasing, and these patients are vulnerable to progression from LTBI to active TB. Therefore, given the possibility of false-negative TST results in the immunocompromised group in our study, the diagnosis of LTBI using TST only could miss a substantial number of candidates for treatment of LTBI infection in South Korea. Some more recent guidelines for the diagnosis of LTBI include a two-step process using the TST followed by IGRA in cases in which the TST result is positive [30]. However, considering the poor compliance with the repeated visit required for two-step TST and IGRA testing, and the considerable number of individuals with TST negative/QFT-IT positive results in the immunocompromised group in our study, the use of both the TST and QFT-IT tests could maximise the efficacy of screening for LTBI in immunocompromised patients.

To fully understand our results, it is necessary to consider the limitations of this study. First, the accuracy of the TST and QFT-IT for the diagnosis of LTBI infection could not be directly estimated in our study because there were no methods to confirm the diagnosis. Second, the study did not include long-term follow-up data for progression to active TB in conjunction with the results of the TST and QFT-IT. Therefore, we could not conclusively demonstrate the superiority of the QFT-IT for the diagnosis of LTBI among immunocompromised patients. Third, no data were included regarding the BCG vaccination status of participants, and therefore the effect of BCG vaccination on the TST and QFT-IT results of immunocompetent and immunocompromised patients could not be analysed. However, because BCG vaccination is mandatory in South Korea, and the age distribution of the two groups was similar, we do not expect BCG vaccination to have greatly affected our results. Fourth, the heterogeneous nature and small number of immunocompromised participants made it difficult to generalize among the various immunocompromised conditions.

## Conclusions

In conclusion, compared with the TST, the QFT-IT assay seems to be more sensitive for detecting LTBI in immunocompromised patients; however, the QFT-IT gave a considerable proportion of indeterminate results among immunocompromised patients. Therefore, the use of both the TST and QFT-IT could maximize the efficacy of screening for LTBI in immunocompromised patients.

## Abbreviations

IGRA: Interferon-γ release assay; LTBI: latent tuberculosis infection; TST: tuberculin skin test; QFT-IT: QuantiFERON-TB Gold In-Tube; TB: Tuberculosis; BCG: Bacille Calmette-Guerin; MTB: *M. tuberculosis; *HIV: human immunodeficiency virus; ESAT-6: early secreted antigen 6; CFP-10: culture filtrate protein 10; QFT ELISA: QuantiFERON-TB Gold enzyme linked immunosorbent assay; OR: odds ratio; CI: confidence interval; BMI: body mass index; ESRD: end-stage renal disease; IQR: interquartile range; WBC: white blood cells.

## Competing interests

The authors declare that they have no competing interests.

## Authors' contributions

EY Kim carried out screening and statistical analysis of the data and participated in the writing of the manuscript. JE Lim carried out screening and acquisition of data. JY Jung participated in the acquisition of data and statistical analysis. JY Son participated in the acquisition of data and analysis and interpretation of data. KJ Lee, YW Yoon and BH Park participated in the acquisition of data, interpretation of data and writing of the manuscript. JW Moon, MS Park and YS Kim participated in the study design and the analysis and interpretation of data. SK Kim and J Chang participated in the study design, analysis and interpretation of data and critical revision of the manuscript for important intellectual content. YA Kang participated in the study design, analysis and interpretation of data and the writing of the manuscript. All authors read and approved the final manuscript.

## Pre-publication history

The pre-publication history for this paper can be accessed here:

http://www.biomedcentral.com/1471-2334/9/207/prepub
